# Spontaneous intestinal bleeding due to pseudoaneurism of the gastroduodenal artery: case report of a rare complication to median arcuate ligament syndrome

**DOI:** 10.1093/jscr/rjaa507

**Published:** 2020-12-18

**Authors:** Kristian K Jensen, Peter Bonde, Jan H Storkholm, Søren T Heerwagen, Peter N Larsen, Jonas Eiberg

**Affiliations:** Department of Surgical Gastroeneterology and Transplantation, Rigshospitalet, Copenhagen, Denmark; Department of Surgery, Bispebjerg Hospital, Copenhagen, Denmark; Department of Surgical Gastroeneterology and Transplantation, Rigshospitalet, Copenhagen, Denmark; Department of Radiology, Rigshospitalet, Copenhagen, Denmark; Department of Surgical Gastroeneterology and Transplantation, Rigshospitalet, Copenhagen, Denmark; Department of Vascular Surgery, Rigshospitalet, Copenhagen, Denmark; Department of Clinical Medicine, Faculty of Health and Medical Sciences, University of Copenhagen, Copenhagen, Denmark; Copenhagen Academy for Medical Education and Simulation (CAMES), Copenhagen, Denmark

## Abstract

Median arcuate ligament syndrome (MALS) is the compression of the celiac artery (CA) by the median arcuate ligament. MALS can cause pseudoaneurysm of the gastroduodenal artery, which can lead to fatal bleeding. A 40-year-old male with no prior medical history presented with symptoms of upper gastrointestinal hemorrhage (UGIH). Severe duodenal bleeding was confirmed although endoscopic hemostasis was impossible and final hemostasis was achieved following a subsequent open duodenotomy. A postoperative computed tomographic angiography (CTA) visualized a significant CA stenosis, post-stenotic dilatation and an aneurysm on a jejunal branch artery. The patient underwent coiling of the gastroduodenal artery, gastroepiploic artery and two pancreaticoduodenal arterial branches. The patient was diagnosed with MALS and 6 months later underwent open resection of the median arcuate ligament. MALS should be considered as a rare cause of upper gastrointestinal bleeding. The literature and proposed treatments are discussed.

## INTRODUCTION

Median arcuate ligament syndrome (MALS) was first described in 1963 and occurs when the celiac artery (CA) is compressed by the median arcuate ligament (MAL) [1]. This condition is seen on computed tomography angiography (CTA) in 10–24%, but in most cases an asymptomatic finding [2]. When causing symptoms consistent with postprandial mesenteric ischemia (postprandial pain, weight loss, epigastric pain during exercise, nausea and vomiting) [3,4], MALS is also known as the Dunbar syndrome [5]. Symptomatic MALS is a rare condition and the clinical significance of CA compression is unclear and the treatment debatable, although laparoscopic resection of MAL has shown promising results in careful selected patients [6]. The prevalence of symptomatic MALS in the population is unknown, whereas the incidence has been estimated to two of 100 000 patients [7]. The syndrome is most prevalent in women (4: 1), in the age group 30–50 years and in patients with a nonobese body composition [7].

We present a rare case with radiological CA compression debuting with severe upper gastrointestinal hemorrhage (UGIH) without the otherwise well described symptoms of MALS.

## CASE REPORT

A 40-year-old male was admitted to the department of surgery due to syncope and melena. The patient had no medical history and no classic MALS symptoms, besides unexplainable weight loss of 12 kg. Due to the weight loss the patient underwent contrast enhanced CT in venous phase 1 year before admission with no pathological findings described.

**Figure 1:**
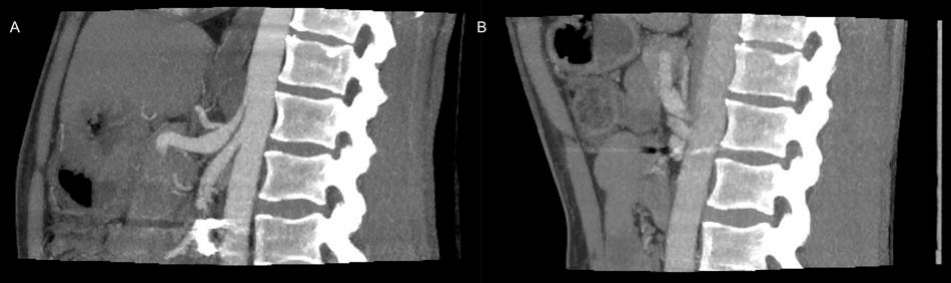
Preoperative inspiratory (**A**) and expiratory (**B**) computed tomography scan, visualizing hook formation and stenosis of the celiac artery.

On admittance the patient had a pulse of 113 BPM, blood pressure of 125/60 mmHg and were anemic with a hemoglobin of 4.7 mmol/L. The patient underwent emergency contrast enhanced CT of both abdomen and cerebrum in venous phase without any pathological findings. Initial upper gastrointestinal endoscopy revealed a pulsating duodenal hemorrhage which could not be controlled endoscopically, and a laparotomy with duodenotomy and surgical hemostasis of a pulsating mucosal artery was performed. No classic duodenal ulceration was seen during neither gastroscopy nor duodenotomy. The patient was discharged 3 days later, but readmitted 6 days postoperatively due hematemesis and syncope. An emergency CTA showed stenosis and post-stenotic dilatation of CA and an aneurysm on a jejunal branch artery. The patient was circulatory stable and transferred to a tertiary center and underwent successful coiling of the gastroduodenal artery, gastroepiploic artery and two pancreaticoduodenal arterial branches. One month later a CTA, following a dedicated MALS protocol, visualized hook deformation of CA during expiration, significant stenosis and post-stenotic dilatation of CA (Fig. 1), all considered pathognomonic for MALS. An additional pseudoanurysm on the pancreaticodorsal artery was identified. The MALS angiographic protocol was performed using contrast-enhanced (100 ml Omnipaque 350 mg/ml, 3 ml/s) arterial scans in a maximum inspiratory phase followed by a maximum expiratory phase was performed with a 5 s delay between the respiratory phases.

The increased collateral retrograde blood-flow in the gastroduodenal and pancreaticoduodenal arteries was suspected to be the underlying reason for pseudoanurism formation and severe UGIH and the patient subsequently underwent laparotomy and resection of the MAL and CA decompression.

### Endovascular procedure

Access was obtained through the right common femoral artery using a 6.5 Fr. steerable sheath (Medtronic Aptus TourGuide), and the CA was catheterized. Subsequently, using a microcatheter system (Progreat 2.7 Fr. (Terumo)) the largest of the two pancreaticoduodenal arcades was catheterized all the way to the inferior pancreaticoduodenal artery, where coiling was started. Detachable microcoils (Concerto Helix (Medtronic) and Interlock-18 (Boston Scientific) were chosen for initial embolization but still dislodged due to the very high inflow in the artery. The coils settled further downstream in the relevant arcade and slowed the flow, thereby allowing for precise deployment of more detachable microcoils in the inferior pancreaticoduodenal artery.

Both arcades, the first few centimeters of the gastroepiploic artery and the gastroduodenal artery (up to the common hepatic artery) were subsequently embolized using multiple nondetachable microcoils (Nester, Cook Medical).

### Open surgical procedure

**Figure 2:**
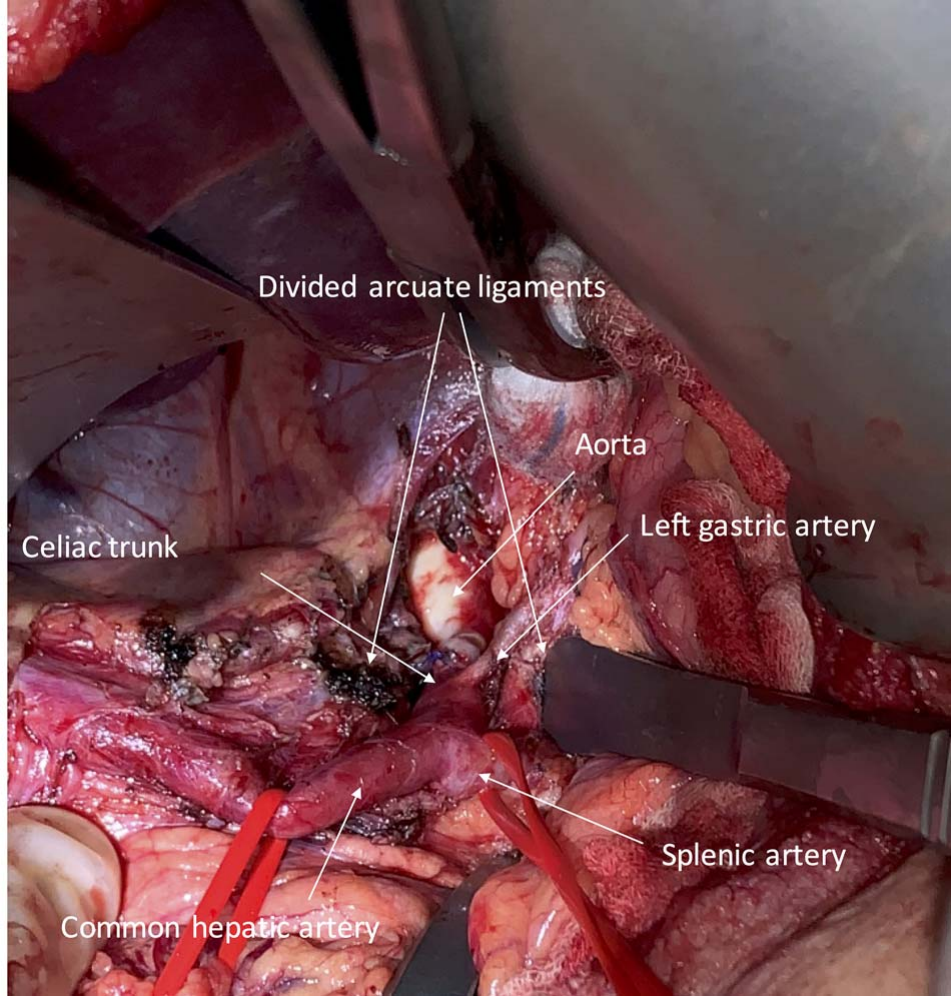
Intraoperative photography showing the exposed celiac trunk and divided arcuate ligaments.

A midline laparotomy was performed and adhesiolysis performed as necessary. Access to the lesser sac was obtained and the CA was identified and followed to the origin at the aorta, where the celiac trunk was found to be tightly compressed by the MAL. All of the muscular fibers of the MAL were divided leaving the origin of the CA completely exposed (Fig. 2). An intraoperative ultrasonography was performed, visualizing a normal flow in the common hepatic artery during both forced in- and expiration.

### Postoperative course

**Figure 3:**
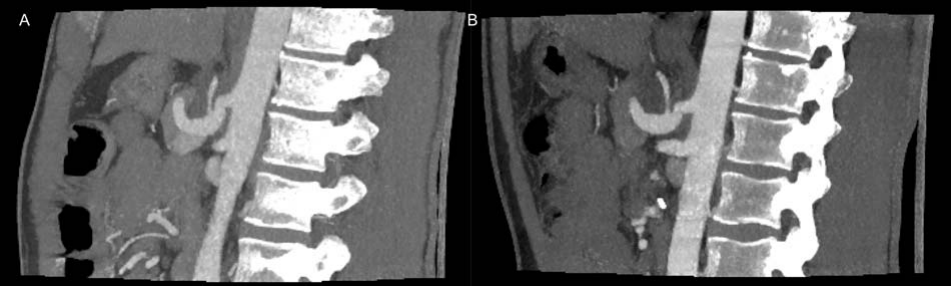
Postoperative inspiratory (**A**) and expiratory (**B**) computed tomography scan with repealed hook formation as well as stenosis of the celiac artery.

The patient was discharged on the third postoperative day, but readmitted 4 days postoperatively due to a minor abdominal wound dehiscence, which was sutured under general anesthesia. On follow-up computed tomography the arterial stenosis was repealed and the pseudoaneurism collapsed (Fig. 3).

## DISCUSSION

The current case describes a severe UGIH most likely secondary to MALS. Compression of the CA by the MAL may lead to stenosis and malperfusion of the upper gastrointestinal tract, and eventually compensatory increased retrograde blood-flow via collaterals and the gastroduodenal and pancreaticoduodenal arteries. Increased collateral flow may cause aneurysm formation, rupture, retroperitoneal hemorrhage and hemorrhagic shock as an extremely rare outcome.

The treatment of the patient in the current case was duodenotomy and surgical hemostasis in the acute phase, which is the treatment of choice in hemodynamically unstable patients with UGIH refractory to endoscopic hemostasis attempts if imminent endovascular service is unavailable [8]. UGIH is associated with a mortality of 6–10%, and the most common cause is peptic ulcer, followed by Mallory-Weiss tear, Dieulafoy lesion and gastroesophageal varices [8]. The selective angiographic embolization was performed as a prophylactic measure of renewed hemorrhage, a treatment that has been proposed as the treatment of choice in comparable literature [9].

The treatment of symptomatic MALS most often is surgical resection of MAL, either open or laparoscopic of the MAL [3, 10]. CA reconstruction, balloon-expandable stenting and robotic MAL division has been sparsely described [6]. We propose that aneurysms of the pancreaticoduodenal arteries are treated with selective endovascular coiling, even in asymptomatic patients, to prevent future massive hemorrhage.

In the emergency presentation of UGIH without peptic ulcer or other common causes, MALS should be considered as a rare underlying pathology.

## CONFLICT OF INTEREST STATEMENT

None declared.

## FUNDING

None.

## References

[1] Harjola PT A rare obstruction of the coeliac artery. Report of a case. Ann Chir Gynaecol Fenn 1963;52:547–50. 14083857

[2] Camacho N Median arcuate ligament syndrome - literature review and case report. Rev Port Cir Cardiotorac Vasc 2017;23:111. 29701344

[3] Kim EN, Lamb K, Relles D, Moudgill N, DiMuzio PJ, Eisenberg JA Median arcuate ligament syndrome—review of this rare disease. JAMA Surg 2016;151:471–7. 26934394 10.1001/jamasurg.2016.0002

[4] Desmond CP, Roberts SK Exercise-related abdominal pain as a manifestation of the median arcuate ligament syndrome. Scand J Gastroenterol 2004;39:1310–3. 15743013 10.1080/00365520410008150

[5] Björck M, Koelemay M, Acosta S Editor’s choice – Management of the diseases of mesenteric arteries and veins: clinical practice guidelines of the European Society of Vascular Surgery (ESVS). Eur J Vasc Endovasc Surg 2017;53:460–510. 28359440 10.1016/j.ejvs.2017.01.010

[6] Kolkman JJ, Geelkerken RH Diagnosis and treatment of chronic mesenteric ischemia: an update. Best Pract Res Clin Gastroenterol 2017;31:49–57. 28395788 10.1016/j.bpg.2017.01.003

[7] Trinidad-Hernandez M, Keith P, Habib I, White JV Reversible gastroparesis: functional documentation of celiac axis compression syndrome and postoperative improvement. Am Surg 2006;72:339–44. 16676860

[8] Feinman M, Haut ER Upper gastrointestinal bleeding. Surg Clin North Am 2014;94:43–53. 24267496 10.1016/j.suc.2013.10.004

[9] Nyman U, Svendsen P, Jivegård L, Klingenstierna H, Risberg B Multiple pancreaticoduodenal aneurysms: treatment with superior mesenteric artery stent-graft placement and distal embolization. J Vasc Interv Radiol 2000;11:1201–5. 11041479 10.1016/s1051-0443(07)61364-5

[10] De’Ath HD, Wong S, Szentpali K, Somers S, Peck T, Wakefield CH The laparoscopic management of median arcuate ligament syndrome and its long-term outcomes. J Laparoendosc Adv Surg Tech A 2018;28:1359–63. 29781769 10.1089/lap.2018.0204

